# Reflecting on One Health in Action During the COVID-19 Response

**DOI:** 10.3389/fvets.2020.578649

**Published:** 2020-10-30

**Authors:** Barbara Häsler, William Bazeyo, Andrew W. Byrne, Marta Hernandez-Jover, Simon J. More, Simon R. Rüegg, Ofir Schwarzmann, Jeff Wilson, Agnes Yawe

**Affiliations:** ^1^Department of Pathobiology and Population Sciences, Royal Veterinary College, Veterinary Epidemiology Economics and Public Health Group, Hatfield, United Kingdom; ^2^Africa One Health University Network (AFROHUN)^‡^, Makerere University, Kampala, Uganda; ^3^One-Health Scientific Support Unit, Department of Agriculture, Food, and The Marine, Government of Ireland, Celbridge, Ireland; ^4^Faculty of Science, School of Animal and Veterinary Sciences, Charles Sturt University, Wagga Wagga, NSW, Australia; ^5^Graham Centre for Agricultural Innovation (NSW Department of Primary Industries and Charles Sturt University), Wagga Wagga, NSW, Australia; ^6^Centre for Veterinary Epidemiology and Risk Analysis, School of Veterinary Medicine, University College Dublin (UCD), Dublin, Ireland; ^7^Section of Epidemiology, Vetsuisse Faculty, University of Zurich, Zurich, Switzerland; ^8^Biosecurity and Food Safety, New South Wales Department of Primary Industries, Orange, NSW, Australia; ^9^Novometrix Research Inc., Moffat, ON, Canada

**Keywords:** SARS-CoV-2, One Health, infectious disease epidemiology, collaborative networks, community network integration, knowledge integration

## Abstract

The COVID-19 pandemic, a singular disruptive event in recent human history, has required rapid, innovative, coordinated and collaborative approaches to manage and ameliorate its worst impacts. However, the threat remains, and learning from initial efforts may benefit the response management in the future. One Health approaches to managing health challenges through multi-stakeholder engagement are underscored by an enabling environment. Here we describe three case studies from state (New South Wales, Australia), national (Ireland), and international (sub-Saharan Africa) scales which illustrate different aspects of One Health in action in response to the COVID-19 pandemic. In Ireland, a One Health team was assembled to help parameterise complex mathematical and resource models. In New South Wales, state authorities engaged collaboratively with animal health veterinarians and epidemiologists to leverage disease outbreak knowledge, expertise and technical and support structures for application to the COVID-19 emergency. The African One Health University Network linked members from health institutions and universities from eight countries to provide a virtual platform knowledge exchange on COVID-19 to support the response. Themes common to successful experiences included a shared resource base, interdisciplinary engagement, communication network strategies, and looking global to address local need. The One Health approaches used, particularly shared responsibility and knowledge integration, are benefiting the management of this pandemic and future One Health global challenges.

## Introduction

The scope and impact of the COVID-19 pandemic is unprecedented in modern times. At the time of writing, over 10 million confirmed human cases and 0.5 million deaths from SARS-CoV-2 infection have been reported (1), and the global community is facing enormous challenges. In these circumstances, an effective response is complex, requiring coherent and collaborative engagement by multiple stakeholders across a diverse network. In a pandemic, a country on its own has limited possibilities, particularly when dealing with a new threat and limited knowledge of its consequences and how to mitigate it; a linking of national priorities and global disease governance is critical (2). For example, knowledge needs to be shared about effective treatments, disease epidemiology including risk factors, people's reaction to measures and effective testing protocols, among others.

One Health is very relevant to the current pandemic. It is concerned with interactions and dependencies in complex systems and promotes a sustainability-oriented approach of health (3) that brings together natural and social sciences and is characterized by collaboration, participation, sharing and exchange in a framework of knowledge integration in health (4). A key feature is the concept of shared responsibility, with the potential for innovative and non-uniform solutions to manage complex problems (5). For example, shared responsibility is used as a collaborative approach to biosecurity management across multiple stakeholders with diverse and complementary perspectives, knowledge and realities to produce robust and prepared biosecurity systems (6). The management and governance of complex biosecurity issues, including prevention, preparedness, detection, response and recovery, is coordinated and shared across government agencies, industry organizations, users and the broader community (7, 8). A clear definition and shared understanding of the concept, including roles and responsibilities, and a consistent and appropriately resourced coordination throughout the system are needed to form true and effective partnerships (9, 10). A shared responsibility approach, agreed upon during peacetime, could support the management of any complex health issue, such as the COVID-19 pandemic, and be implemented at different levels (local, regional, national and international).

The implementation of a partnership approach does not come without its challenges. Knowledge integration and particularly the sharing of data is impacted by political boundaries, as shown in previous evaluations of One Health initiatives (11). Further, sectoral and disciplinary silos constitute an impediment to the ability of stakeholders to mount a timely and effective outbreak response. In such systems, there is potential to improve the efficiency of information flow and knowledge exchange and integration. Traditionally, in the various health sectors, solutions are often prescribed top-down, implying singular linear pathways in isolated aspects of health, whereas health agency and shared responsibility approaches may be more suitable when dealing with unpredictability, uncertainty, and ambiguity.

A recent promising approach to support such collaborative approaches and implement shared responsibility in practice is called Community Network Integration. It aligns distributed networks under a common leadership and collaborative governance framework including means to identify and engage appropriate expertise, human resources and co-funding in order to execute priority scalable solutions-oriented (pilot) projects. The approach also integrates a systems approach to project management, communication, and data integration as well as novel application of principles of social psychology to engage stakeholders and create a culture of high emotional energy vital to collaboration and creative problem solving (12). Thereby, it operationalises the essential dimensions of One Health that include (1) systemic thinking, (2) holistic planning, and (3) transdisciplinary working, supported by an enabling environment to allow for (4) sharing, and (5) learning, endorsed through (6) a systemic organization (13).

In this article, we use three case studies from different world regions to discuss elements of One Health approaches in the COVID-19 response. The three case studies are based on the authors' experiences and illustrate which of the essential One Health dimensions listed above applied in practice during the crisis. They provide examples of collaboration, shared responsibility and knowledge integration and illustrate opportunities and weaknesses.

## Case Studies

### Case Study 1: COVID-19 Modeling Support in the Republic of Ireland: A Case-Study of Rapid Response Demonstrating the Value to Utilizing Cross-Disciplinary Actors Toward a Common Goal

In Ireland, the National Public Health Emergency Team (NPHET) was established on 27 January 2020, to provide national direction, support and expert advice on the development and implementation of a strategy to contain COVID-19 (14). The first confirmed COVID-19 case in Ireland was reported on 29 February, the Special Cabinet Committee on COVID-19 was formed on 3 March, and a National Action Plan was published on 16 March.

NPHET was supported by a number of expert groups, including the Epidemiological Modeling Advisory Group (IEMAG), which was established on 7 March [(15); see Supplementary Figure 1]. IEMAG was tasked with developing capacity for mathematical modeling (epidemiological, demand/supply, geospatial) to enable real time modeling of COVID-19 in the Irish population, drawing on expertise in relevant disciplines from government agencies and universities throughout Ireland. Here we focus on the epidemiological parameters team within the IEMAG epidemiological modeling subgroup, which was tasked with gathering evidence on key characteristics of COVID-19. An important remit of the team was to link biological understanding with technical quantitative skills to improve the building of mathematical and statistical models and help communicate effectively the findings to NPHET and other stakeholders.

The requirement for a rapid response led to a broad call to action from stakeholders with various expertise to contribute, in some cases beyond the traditional human medical disciplines. The team was chaired by a veterinary epidemiologist, with interdisciplinary membership from human public health, agriculture, veterinary medicine, food safety, disease ecology, and One Health backgrounds. Initial team selection was guided by disciplinary expertise, full-time availability (at short notice) and prior working relationships. The group's diverse interests and skills were well-suited to rapidly gathering evidence, and undertaking quantitative secondary and meta-analyses, in response to the emerging threat (16–22). In the context of IEMAG, the multidisciplinary One Health team were able to ensure that the national mathematical models were underpinned by robust biological understandings, both during model development and evaluation. This was particularly important in the context of model fitting to emerging datasets, where the evidence base, and basic understanding of the epidemiology of the pathogen, was rapidly changing. Due to the rapid and changing needs of modelers, the composition and focus on tasks by the subgroup was dynamic, with members requiring to pivot from one parameter to another. In addition, the expertise and experience of the national Health Information and Quality Authority (HIQA), and researchers with particular skills (e.g., virology) were sought and contributed to the network, as required. Throughout, advice from international expertise (e.g., World Health Organization, European Center Disease for Disease Prevention and Control) were monitored and incorporated into IEMAG's work.

In terms of lessons learned, the rapid community-based aggregation of skills applied to a single acute problem should be held as an exemplar of how a distributed network of expertise can contribute in an efficient and effective way toward a goal. Interdisciplinary synergies were central to progress, both between mathematics and the life sciences and, importantly, between medical and allied disciplines. One Health perspectives predominated and there was cross-pollination of ideas and skills across disciplines to achieve efficiencies and better, more dynamic, systems. Challenges included remote working while maintaining communication and ensuring there was no duplication of effort across various NPHET subgroup teams. Furthermore, given the finite resources available, the COVID-19 response led to a temporary diversion of expertise and resource from other aspects of national animal health management. This case study is an important example of new thinking, diversity of thought, and new networks of expertise within Ireland.

### Case Study 2: A State Level One Health Approach to Respond to COVID-19: Perspectives From the New South Wales Department of Primary Industries

On 21 January 2020, the Australian Chief Medical Officer (CMO) issued a determination adding “human coronavirus with pandemic potential” to the Biosecurity (Listed Human Diseases) Determination 2016. As a result, the Australian Health Protection Principal Committee, the key decision-making committee for health emergencies formed by the CMO and state and territory Chief Health Officers, was convened and daily meetings activated. In addition, national coordination was also activated for responding to the health emergency through the National Incident Room, the strategic reserve of personal protective equipment through the National Medical Stockpile and the provision of clinical and academic leadership through the National Trauma Center. Meetings of state, territory and Commonwealth health ministers to discuss pandemic readiness also started. On 25 January, Australia reported its first case of COVID-19, and the Australian Health Sector Emergency Response Plan for Novel Coronavirus (COVID-19 Plan) was implemented on 7 February (23). The COVID-19 Plan acknowledges that the primary responsibility for managing the impact of the outbreak lies with the state and territory governments (24). In New South Wales (NSW), the NSW State Emergency Management Plan (EMPLAN) and the NSW Human Influenza Pandemic Plan (sub-plan to EMPLAN) were implemented (24, 25).

Early in the response, a One Health approach was implemented through the collaborative engagement of animal health experts, including veterinarians and epidemiologists, from NSW Department of Primary Industries (DPI) and other institutions (e.g., universities, consultants), sharing expert knowledge. This approach is pre-defined by EMPLAN, under which a Combat Agency is nominated to lead operations (in this case, the Ministry of Health) and able to request support from other government areas, such as the NSW DPI. Animal health specialists worked for the Public Health Emergency Operations Center, responsible for activities such as tracing, research and providing expert advice, in the epidemiology and tracing units. In addition, the Ministry of Health liaised with other government agencies to establish remote tracing capabilities, including sharing of databases, online training and debriefs (due to the travel limitations) and the need to increase contact tracing capacity. As the responsible agency for providing agriculture and animal support during emergencies (under EMPLAN), NSW DPI as the Agriculture and Animal Services Functional Area was present within the State Emergency Operations Center throughout the response, liaising with health services with respect to animal care. Furthermore, the NSW state animal laboratory provided diagnostic services to NSW Health. NSW DPI worked with Australia's Animal Health Committee (AHC) to develop science-based, nationally consistent policy on animal health issues related to COVID-19, and supported the agriculture and animal sectors in achieving continuity of their businesses to safeguard animal health and welfare and help ensure a secure food supply (26). AHC developed and implemented policies, operational strategies, risk assessments and communications around SARS CoV-2 and animals and managing Emergency Animal Diseases (EAD) during human pandemics.

As key learning of this response, COVID-19 highlighted the importance of a well-resourced response using a One Health approach, involving a broad range of human and animal health stakeholders and shared resources, which could then be scaled back as needed. The COVID-19 situation also highlighted the need for appropriate communication and management of animal health and welfare during human pandemics.

### Case Study 3: One Health in Action: Experiences From the Africa One Health University Network (AFROHUN) COVID-19 Knowledge Sharing Response

The Africa One Health University Network (AFROHUN), formerly One Health Central and Eastern Africa (OHCEA) is a University led network of 24 public health, veterinary medicine, pathobiology and environmental health institutions and 16 universities in eight countries in East, Central and West African regions (Cameroon, Democratic Republic of Congo, Ethiopia, Kenya, Rwanda, Senegal, Tanzania, and Uganda). Since its inception in 2010, AFROHUN supports institutional changes in teaching and learning environments in higher institutions that promote One Health approaches.

In the absence of a global workforce, most African national COVID-19 response actions relied on national health professionals to provide the much-needed workforce in the management of the pandemic. Universities were among the key institutions that supported different national response task forces. University members served on scientific task forces with evidence-based and science-based data shaping response strategy options as the mainstream health workforce within ministries were at the forefront of the response.

Between 23 March 2020 and 18 June 2020, AFROHUN through its wide continental network in collaboration with the USAID-funded One Health Workforce – Next Generation (OHW-NG) consortium led by University of California, Davis, provided a platform where network members (faculty and students), practitioners in One Health, and stakeholders virtually via ECHO (Extension for Community Healthcare Outcomes^1^) sessions twice a month accessed the current information on COVID-19 as it evolved. Expert presentations were made by global and in-country teams and real issues and dynamics experienced during response actions were discussed in an interactive way. Selected topics for discussions were delivered over three months by experts in infectious disease epidemiology, human medicine, public health, environment and occupational health, veterinary medicine, immunology and molecular biology, among others, working at the forefront of the response at country, regional and global levels. This provided expert knowledge and experiences on COVID-19 to faculty and practitioners during the webinars. The knowledge gained from the webinars was appreciated by participants, some of whom used it in their different roles in national COVID-19 response teams while a number of faculty indicated readiness to use the rich knowledge in their classes when teaching students. The sessions were perceived to provide valuable knowledge that participants used in their national duties on different COVID-19 task forces, as illustrated by these quotes:

“*During this period, we were discussing options to reshape response measures in the surveillance commission because in Kinshasa capital city cases were still rising. At that time, the herd immunity theory that was discussed during the AFROHUN COVID-19 session on immunity issues and interventions for COVID-19. We learned more about it and about the advances in vaccine development. This improved my knowledge, which I shared, and helped us to focus on improving our testing capacities, as there is no evidence supporting such a theory*.” Dr. Marc Yambayamba, AFROHUN country manager in DRC, member of the national COVID-19 surveillance team.

“*Based on knowledge we have gained on multidisciplinary approaches in addressing health issues, we mobilized students into One Health Student Club (SOHIC) and we have been active in COVID-19. The club, a multidisciplinary team of students from Makerere University and Mbarara University of Science and Technology in Uganda have been raising awareness about COVID-19 to communities and providing mental health support.”* Muganzi David Jolly, President, Students One Health Innovation Club, Mbarara University of Science and Technology, Uganda.

“*I was asked to lead a team that was responsible for advising the government on the design and necessity for wearing cloth face masks in crowded places such as bus stands, markets, hospitals and places of warship. Now mask use is widespread as one of the preventive actions against COVID 19. In my leadership role, I have used some of the ideas from the AFROHUN ECHO sessions.”* Prof. Japhet Killewo, Professor of Epidemiology at Muhimbili University of Health and Allied Sciences (MUHAS), Tanzania.

An AFROHUN internal review identified several lessons learnt from the three months of COVID-19 sessions. The power of existing platforms, strong leadership, the combination of global and in-country perspectives and the ability of leveraging networks was highlighted, with around 200 participants and experts being part of the sessions, providing valuable multi-disciplinary, global and local in-country perspectives. The tight schedules of the task force members at the frontline of the pandemic prevented engagement of mainstream ministries in the design of the sessions. Participation of representatives from government and members from different COVID-19 task forces helped bridge this gap. Their perspectives on the issues and dynamics of the pandemic helped to shape subsequent sessions. A more efficient information flow in the national response system to reach diverse users could have enhanced the design and delivery of the sessions. Official engagement of specific task forces such as the scientific committees on the ECHO sessions could have added value.

## Discussion

The three case studies each demonstrate important benefits from the use of One Health approaches in the management of the COVID-19 pandemic. The sharing of resources, multidisciplinary engagement and communication network strategies were common across the three case studies, in support of knowledge integration and more effective response management. Each can be placed within a One Health framework (13), providing examples of an effective engagement of expertise and in-kind resources (e.g., labor, connectivity, materials) from a broad range of relevant stakeholder groups. Moreover, they illustrate One Health approaches within inclusive national and local outbreak teams, including transparent use of information, multi-way dialogue, information sharing, and the development of solutions through collaborative learning.

With regards to the six One Health dimensions that are described at the beginning of the paper, the three case studies all had clear sharing and learning structures in place that facilitated an exchange of data, information, and knowledge, as well as accessing and generating new knowledge through collaborative processes. All case studies described working across disciplines, but remained within the boundaries of the natural sciences and did not engage either the social sciences or the humanities. Also, wider society engagement was lacking, which meant that collaborative working remained within the multi- and interdisciplinary spheres and did not reach transdisciplinary working. Holistic planning was a key feature of case study 2, which provided a strong basis for the actions implemented. Systemic organization was dominant for AFROHUN, with the existence of a large, formal network of universities that allowed prompt recruitment of scientific experts into the response. None of the case studies explicitly described systemic thinking even though it is advocated by the WHO (27) and the Association of Schools and Programs in Public Health (28, 29). These case studies were conducted during emergency situations where rapid and unequivocal instructions and responses are demanded. In contrast, system thinking requires that the problem is adequately formulated, the right stakeholders are selected, a vast set of problem-solving options are considered, boundaries are defined correctly, the approach is systematic rather than focussed, and connections are not ignored. Given the need for rapid responses, the resulting “messiness” introduces uncertainty, and may unearth conflicts in ethics, values, judgement and background experiences. In addition, perspectives may change due to system dynamics which pose additional challenges to public communication (30). This may emphasize that these debates must take place as part of the preparedness process if they should be operational in an emergency situation.

The case studies have demonstrated how expertise can be mobilized and shared quickly, given appropriate support infrastructure and in the light of the pressing needs of the pandemic. It is hoped that lessons learned can be extended to “peacetime,” outside the crisis. Interdisciplinary synergies, underpinned by One Health concepts, will also be needed to manage critical global challenges, including those relating to climate change and antimicrobial resistance (31). To harness both the power of new thinking and networks of expertise, it is recommended that preferred solutions are supported by effective network-wide business systems (e.g., management, financial, communications, IT, and human resources) and dynamic learning facilities conducive to transparent knowledge and data sharing, dialogue and innovation.

## Data Availability Statement

The original contributions presented in the study are included in the article/Supplementary Material, further inquiries can be directed to the corresponding author/s.

## Author Contributions

BH, AB, and MH-J conceived the study. AB and SM wrote the case study 1 (Ireland). MH-J and OS wrote the case study 2 (NSW, Australia). WB and AY wrote the case study 3 (AFROHUN). All authors contributed to developing, writing, editing of the paper, and approved the manuscript for publication.

## Conflict of Interest

JW was employed by the company Novometrix Research Inc, a social enterprise which facilitates alignment of diverse stakeholders for solutions in economic, social and environmental sustainability. JW and Novometrix Research have no financial interest in any aspect of this manuscript. The remaining authors declare that the research was conducted in the absence of any commercial or financial relationships that could be construed as a potential conflict of interest.

## Footnotes

^1^ECHO model™, https://echo.unm.edu/about-echo/model/

## Supplementary Material

Supplementary Figure 1 Overview diagram of the structure of the National Public Health Emergency Team (NPHET), including the broad stakeholder composition demonstrating the interrelationship between government, public health authorities, and academia in response to the COVID19 epidemic in Ireland. CMO, Chief Medical Officer.
