# Supplementary figures and images for: Reflecting on One Health in Action During the COVID-19 Response
Source: Front Vet Sci. 2020 Oct 30;7:578649. doi: 10.3389/fvets.2020.578649 (PMC7661772; doi:10.3389/fvets.2020.578649)

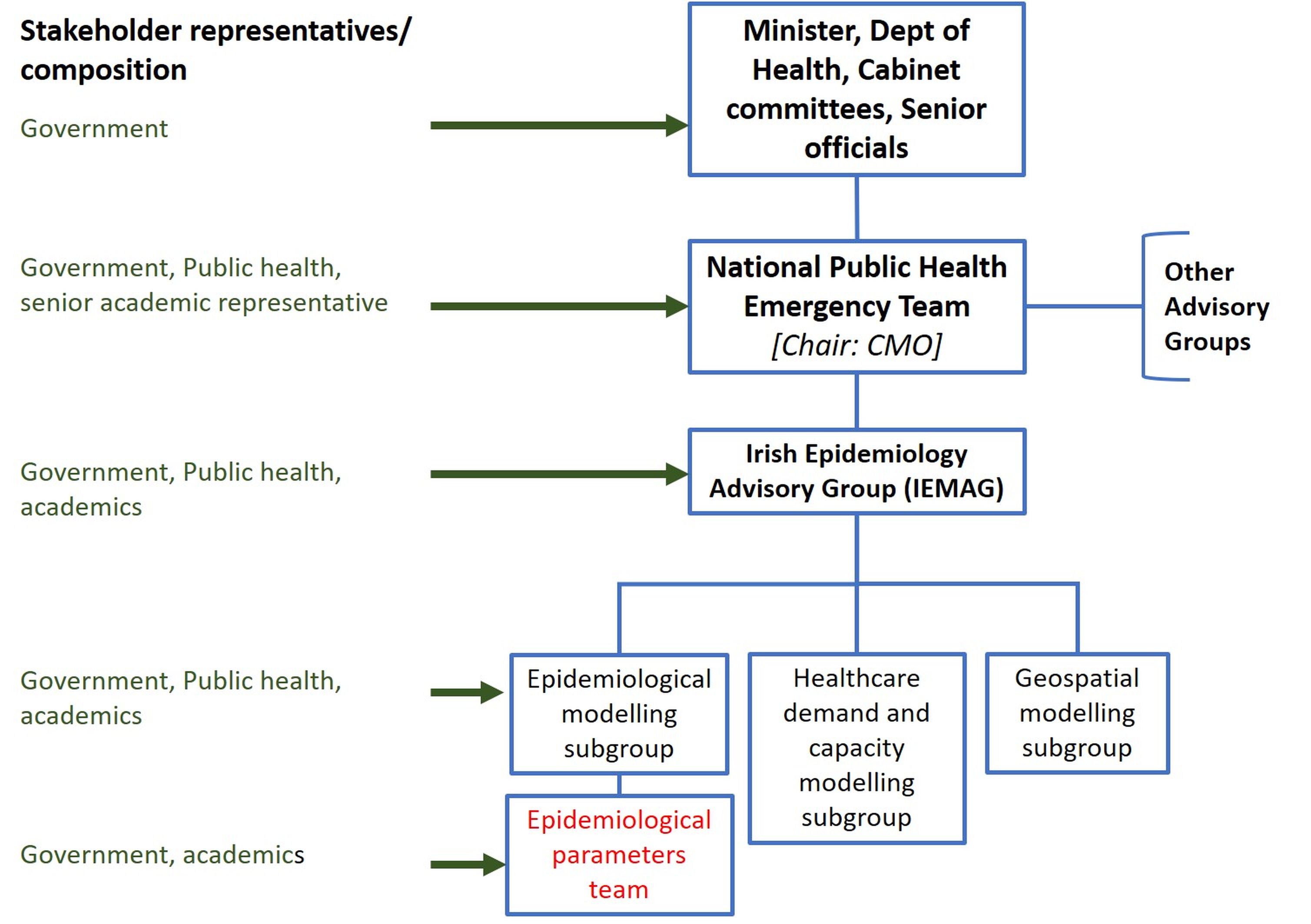

Supplement: Supplementary Figure 1 — Overview diagram of the structure of the National Public Health Emergency Team (NPHET), including the broad stakeholder composition demonstrating the interrelationship between government, public health authorities, and academia in response to the COVID19 epidemic in Ireland. CMO, Chief Medical Officer. [file Image_1.JPEG]
